# Major remaining gaps in models of sensorimotor systems

**DOI:** 10.3389/fncom.2015.00070

**Published:** 2015-06-04

**Authors:** Gerald E. Loeb, George A. Tsianos

**Affiliations:** ^1^Department of Biomedical Engineering, University of Southern CaliforniaLos Angeles, CA, USA; ^2^L-3 Applied Technologies Inc., HEM DivisionSan Diego, CA, USA

**Keywords:** sensorimotor control, sensorimotor learning, sensorimotor integration, sensorimotor systems modeling, biological neural networks

## Abstract

Experimental descriptions of the anatomy and physiology of individual components of sensorimotor systems have revealed substantial complexity, making it difficult to intuit how complete systems might work. This has led to increasing efforts to develop and employ mathematical models to study the emergent properties of such systems. Conversely, the development of such models tends to reveal shortcomings in the experimental database upon which models must be constructed and validated. In both cases models are most useful when they point up discrepancies between what we think we know and possibilities that we may have overlooked. This overview considers those components of complete sensorimotor systems that currently appear to be potentially important but poorly understood. These are generally omitted completely from modeled systems or buried in implicit assumptions that underlie the design of the model.

## Introduction

“When you can measure what you are speaking about, and express it in numbers, you know something about it, when you cannot express it in numbers, your knowledge is of a meager and unsatisfactory kind; it may be the beginning of knowledge, but you have scarcely in your thoughts advanced to the stage of science.”

—William Thomson, 1st Baron Kelvin, 1883

Quantitative methodology has gradually replaced qualitative “butterfly collecting” in biology. Lord Kelvin's comment was motivated primarily by its importance for reductionistic science, whereby hypotheses about deep and unobservable structure and function can be tested according to their often subtle effects on measureable phenomena. More recently, the challenge of science has more often been too much rather than too little quantitative data, and too many well-understood but complex mechanisms whose interactions defy intuitive understanding of how complete systems actually function. This problem combined with the rapid advance of computing power has led to rapidly increasing interest in systems modeling. For the 21st century, one might replace “measure” with “model” to update Lord Kelvin's 19th century exhortation.

Modeling is a never-ending task. It is most useful when it reveals discrepancies between what we think we know about a component or a complete system and how it actually behaves experimentally. The existence of a model (whether explicit or implicit) is an inspiration to experimentalists to identify phenomena or conditions for which the model predictions are in error—the basic scientific method of hypothesis formation and falsification. Assuming the experimental results are valid, the discrepancy can only be resolved by correcting and usually complexifying the model to reflect better the properties of the physical system being modeled. As more experimental data become available, it becomes possible, indeed necessary, to break complex systems into subsystems that can be studied and modeled in relative isolation. This leads to a proliferation of models of subsystems that must eventually be combined but that may be at very different stages of development and accuracy. This problem is amplified by the natural tendency of scientists to focus on and further refine those subsystems that have already yielded to their efforts. Put simply, we have a lot of quantitative information incorporated into accurate models of some subsystems but little or none about other subsystems that are likely to be just as important to overall function or about most of the connections between subsystems.

**Figure 1:**
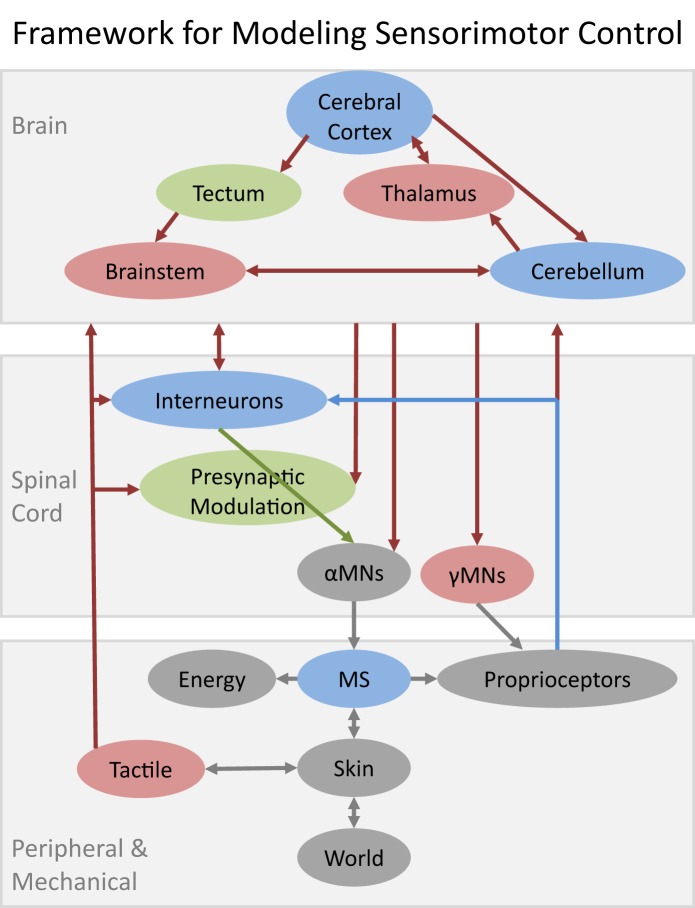
**Schematic of major structures likely to be required for a reasonably complete model of sensorimotor control of learned voluntary behaviors**. Gray elements denote well-modeled subsystems (discussed herein only by reference); blue denotes substantial progress but with important functional gaps; green denotes attempts to model based on incomplete data; red denotes no quantitative models to date. Musculoskeletal systems (MS) interact with the World via Skin interfaces that contain large numbers of Tactile mechanoreceptors. Other sources of neurally mediated feedback include large numbers of Proprioceptive mechanoreceptors in muscles and various connective tissues plus chemoreceptors that provide information related to Energy consumption, fatigue, and injury. All of these somatosensory signals inform both the spinal cord and the brain and are known to play critical roles in both learning and performance of skilled motor behaviors. Various computational models have been described for individual subsystems of the brain but these models do not include specific models of how they interact with each other or with their ascending and descending projections from and to the various subsystems in the spinal cord.

The nature of the modeling challenge depends on where a subsystem is located in a system that is inherently hierarchical (Figure 1). The subsystems in the spinal cord and musculoskeletal system have mostly been studied in isolated or highly reduced preparations. Such subsystems are amenable to “bottom up” modeling strategies in which individual elements are characterized and then combined into larger models. Shortcomings in the constituent models tend to arise because it is difficult for the experimenter to observe or create the full range of natural conditions of use. The subsystems in the brain have been studied mostly in intact, naturally behaving animals. Those subsystems are generally modeled using “top-down” strategies in which the form of the model is intuited from observable phenomena of the whole system. Shortcomings in those models tend to arise because the experimenter must make assumptions about what is or is not happening in the other subsystems that are present. This overview is not an encyclopedic review of what models already exist. Rather it attempts to prioritize those subsystems that currently appear to be both important and relatively poorly modeled and to identify opportunities to rectify this.

## Systems model architecture

“It can scarcely be denied that the supreme goal of all theory is to make the irreducible basic elements as simple and as few as possible without having to surrender the adequate representation of a single datum of experience.” (Frequently paraphrased as “A scientific theory should be as simple as possible, but no simpler.”)

—Albert Einstein, 1933

Biological systems (and their models) tend to be much more inherently complex than the physical systems that Einstein had in mind. This is an inevitable consequence of their gradual evolution over hundreds of millions of years of intense competition with other species. Relatively simple mechanisms such as actin-myosin binding for force generation and a stretch reflex to stabilize posture can have their essence captured by mathematical curve-fitting (Hill's equation) and engineering metaphor (servo-control), respectively. Real organisms, however, achieve their competitive performance by huge and disorderly elaborations of those underlying mechanisms. This poses a potential conflict of interest between the modeler, who often aspires to simple and elegant models, and the experimentalist, who needs to capture realistic performance.

The modeler must decide what performance is to be modeled, thereby defining the functional elements that must be included in the model system. This is itself a form of modeling that is fraught with opportunities for errors of omission. Figure 1 provides one framework for the restricted set of learned, voluntary sensorimotor behaviors such as manipulating objects. Smaller and simpler models have been used with substantial success to account for preprogrammed behaviors such as locomotion, breathing, and mastication. Larger and more complex models will be required to account for multimodal behaviors such as eye-hand coordination.

The framework in Figure 1 can be divided hierarchically and phylogenetically into brain, spinal cord and peripheral/mechanical subsystems. The first organisms to achieve motility evolved by steadily enhancing their abilities to make movements that were environmentally responsive, mechanically stable, and energetically efficient. Sensory transduction and electromechanical activation were already well on their evolutionary paths before there were recognizable nervous systems at all. As organisms became larger and more complex mechanically, coordination required centralization of sensorimotor connections in invertebrate ganglia that eventually coalesced into the vertebrate spinal cord. Systems-level modeling is often applied to behaviors that are learned by the brain but implemented by the “lower” but highly evolved subsystems, whose intrinsic properties thereby define the control problems that the brain must solve. Model systems that omit or substantially simplify components are implicitly hypothesizing that those components do not make a significant contribution to the observed behavior, even though they are known to be necessary and perhaps even sufficient for many other behaviors of the same organisms. Unfortunately, such omissions more often reflect the unavailability of computational models rather than such a plausible hypothesis.

Explicit or implicit models that have been incorrectly simplified lead to progressively more complex and implausible models as they try to account for new data. This is analogous to the problem of “the music of the spheres” in which an intuitive and simple earth-centered universe requires ever more artificial structure to account for the observed motion of the planets. Such a problem may be starting to emerge in motor learning models that assume that the motor cortex is directly responsible for converting visual targets in extrapersonal space into sequences of muscle activation that cause limbs to reach to those targets. Such models often assume that the cortex learns an “internal model” of the musculoskeletal plant and inverts that model to compute the commands required to perform a given task. As Nikolai Bernstein pointed out (Bernstein, 1967) (English translation of 1934 publication in Russian), such a computational problem is ill-posed because of redundancy. There are usually more muscles and degrees of freedom than required to perform the task, so the computation requires either an arbitrary constraint on allowable strategies (d'Avella et al., 2006) or an optimization criterion such as minimizing effort that will result in a singular solution (reviewed in Loeb, 2012). If there were a single internal model in a single place subject to a single computational strategy, then one would expect this to give rise to rather consistent patterns of sensorimotor adaptation and learning. Experiments on learning and adaptation instead reveal many different “rules” whereby subjects learn and forget how to deal with distortions in the visual space, loads on the limb, and changes in posture and muscle function and performance criteria, as well as interactions among those variables (see Shadmehr and Mussa-Ivaldi, 1994; Gandolfo et al., 1996; Krakauer et al., 2000, 2006; Baraduc and Wolpert, 2002; Mattar and Ostry, 2007, 2010; Pearson et al., 2010; Brayanov et al., 2012; Berniker et al., 2014). Substantial evidence has shown, however, that internal representations of behavior are neither intuitive nor simple (Brayanov et al., 2012) and that sensorimotor learning generalizes poorly in many situations (Gandolfo et al., 1996; Mattar and Ostry, 2007, 2010; de Rugy et al., 2012b; Coelho et al., 2013; Berniker et al., 2014), which is inconsistent with the predictions of a singular internal model. If these behaviors were actually the result of several different sensorimotor subsystems, each with their own relatively simple rules, the inferred models might actually be simpler as well as more realistic. Such a collection of interacting subsystems is known to subserve control of gaze, which consists of anatomically distinct subsystems for classes of behavior such as voluntary and involuntary saccades, smooth pursuit, and reflexive stabilization, and the coordination of eye and head movement to achieve them. The anatomical structures responsible for gaze control include the mesencephalic tectum, pontine, and other brainstem nuclei and cerebellum as well as sensory and motor cortical areas, all of which also have strong sensory and motor connections with the limbs.

## Gaps in peripheral and mechanical models

Models of the musculoskeletal system include several different types of models that must be combined to generate their complete input/output properties.

### Musculoskeletal dynamics

Control engineers divide any system into a *plant* and a *controller* and describe the tasks to be performed using *cost functions* that weight the relative importance of quantifiable *state variables* such as accuracy, time to completion and energy consumption. The job of the sensorimotor nervous system (*controller*) is to compute and implement control signals that cause the musculoskeletal system (*plant*) to achieve desired performance. The mechanical dynamics of the plant obviously constrain the set of useful solutions for the controller. More complex plants make it more difficult for engineers to develop mathematical models that can be used to compute solutions, but they do not necessarily make the plant inherently more difficult to control. For example, the complex intrinsic properties of muscles (e.g., dependency of force output on instantaneous muscle length and velocity) may provide rapid stabilizing effects that compensate in part for the relatively long delays inherent in signal transmission in a neural controller (Hogan, 1984; Loeb et al., 2002) but see (Crevecoeur and Scott, 2014). Nevertheless, the mechanical dynamics of multilinked skeletal segments such as a limb or vertebral column are inherently complex and can result in highly unstable conditions that must be prevented by a controller with widely distributed inputs and outputs (Lackner and DiZio, 2000).

Sophisticated algorithms for solving the mechanical dynamics of free-body systems with mechanical constraints have been widely applied to the biomechanics of musculoskeletal systems. These work reliably when the biological architecture lends itself to decomposition into discrete and independent entities—the inertial segments, joints, and actuators typical of most limbs. Mechanical modeling becomes difficult and less reliable when the discrete entities interact through distributed connective tissues. In the hand, this arises when tendons from multiple muscles insert onto capsular structures around finger joints rather than directly onto individual bones and when separately controlled neuromuscular compartments of multiheaded digit muscles are loosely coupled to each other (Schieber and Santello, 2004). Distributed viscoelasticity in the skin tends to dominate the mechanics of the lightweight digits at low muscle recruitment. In the neck and trunk, modeling challenges arise when the length and/or pulling direction of one muscle depends on the position or activation of other muscles (Richmond et al., 2001). In the shoulder and hip, this occurs when muscles wrap over and around other muscles (van der Helm, 1994). In theory, these complexities can be approximated by decomposition into multiple, discrete and classical entities. In practice, most of the parameters that need to be specified to define such finite element models are unavailable.

Models of the rapidly conducting proprioceptors (myelinated nerve fiber groups I and II) are well-developed but they must be driven by perhaps uncertain musculoskeletal dynamics (muscle fascicle length and velocity for spindles and active muscle force for Golgi tendon organs). Several other types of mechanoreceptors have been described in muscles, ligaments, and joint capsules (Grigg and Hoffman, 1984), but mathematical models are not available and their functional roles are uncertain.

### Non-stationarity of muscle physiology

Models of the contractile properties of mammalian skeletal muscle are perhaps the most developed of all components. They were developed initially to explore reductionist models of contractile mechanisms under highly limited and generally unphysiological operating conditions (e.g., isometric or isotonic twitch or tetanus). They were extended to account fairly well for the full range of kinematic conditions and contractile properties of mammalian slow-twitch and fast-twitch muscle fibers at various physiological levels of recruitment. More recently they were extended to account for energy consumption (Tsianos et al., 2012), which may be an important cost-function used by the brain to improve performance during motor learning. What is missing are models of how the properties of muscles change over time as a result of patterns of use or disuse. The control strategies learned by the brain must anticipate or at least cope well with these changes. Understanding such changes is important when modeling is used to account for performance in pathological systems (see below).

In the short term, muscles are subject to fatigue—a reduction in force output for a given set of operating conditions (Enoka and Duchateau, 2008). Such reductions may arise from changes in the many cellular processes involved in muscle activation and deactivation. Models of muscle that are composed of computational elements that correspond to the energy-consuming processes (e.g., cross-bridge turn-over and calcium flux) should make it possible to account for those changes, but this remains to be modeled. The effort will require a great variety of experimental data, much but perhaps not all of which is already available in the literature. Many diseases and injuries are associated with disuse atrophy of muscles, which tends to increase greatly their susceptibility to fatigue, but the relative contributions of the underlying processes may be different from normal muscles.

Musculotendinous injuries account for the majority of emergency medical treatment and long-term disabilities, so there is a great deal of interest in how they occur, how they affect performance and how they heal. Structural engineering has benefitted greatly from finite-element analytical modeling (FEM) of complex structures. There have been a few attempts to develop such models for muscle, which is essentially a composite material consisting of contractile elements (muscle fibers) embedded in a structurally critical matrix (endomysial collagen) (Trotter, 1993).

Muscle is particularly responsive to exercise, which results in rapid and profound changes in key properties such as force-output, speed of contraction and relaxation, and resistance to fatigue. Quantitative models of these changes are becoming more feasible as the cellular mechanisms responsible for signaling and managing plasticity are starting to be revealed. Data from formal physiological experimentation and the effects of athletic training tend to emphasize covariances in physiological properties; e.g., intense but brief exercise tends to develop fast-twitch muscle fibers with larger force output, faster rise and fall dynamics, higher rates of maximal shortening (V_max_) and lower fatigue resistance than slow-twitch muscle. Mathematical models that represent explicitly the mechanisms underlying these properties are a better starting point for models of plasticity because the properties do not necessarily covary. Little systematic data are available about the rates at which the individual properties other than maximal isometric force change during normal training. For example, muscles subject to disuse atrophy produce lower force (typical of slow twitch fibers) but also have lower fatigue resistance (typical of fast twitch fibers).

The anatomy and physiology of mature neuromusculoskeletal systems are the result of myriad mechanisms that orchestrate their development (Crawford and Horowits, 2011). Most musculoskeletal models assume that the pinnation and sarcomere lengths of muscle fascicles tend to be optimized for the range of lengths that the muscles experience during normal function and that the connective tissues that support them are matched to the stresses that the contractile elements apply during those functions. The trophic factors that drive the deposition and structural properties of collagen in tendons, aponeurosis, and endomysium are starting to be understood qualitatively but quantitative models of their dynamics are not yet available. Assumptions of covariance and optimality are likely to breakdown if models are applied to pathological conditions. For example, passive tensile properties of mammalian muscle are dominated by the endomysial connective tissue, so do not necessarily covary with active tension or force-length properties that are determined by the myofilaments (Brown et al., 1996).

### Tactile mechanics and sensory transduction

Musculoskeletal systems interact with the world via contact regions composed of skin and related epidermal structures (nails, claws, teeth, hair, or fur). The mechanical compliance of skin defines what happens during object manipulation, which is essentially a series of collisions between the object and various parts of the hand; the resulting deformations of the skin define what information will be available to the CNS from tactile mechanoreceptors, which are all essentially strain gauges. The mechanical properties of these interfacial regions are starting to be captured by finite element modeling (Kumar et al., 2015). Such models can be added to classical free-body models to describe accurately the mechanical events that occur during collisions and manipulation between, for example, fingertips and objects to be grasped, but the computational load is often daunting. Modeling the tactile sensory signals that will result from myriad, independent receptors in the soft tissues remains challenging. Such sensory signals are known to be essential for dexterity, which is severely impaired when skin is anesthetized while proprioception and motor commands are left intact. The histological structure and physiological responsiveness of the receptor modalities are fairly well-understood, so it should be possible, albeit computationally challenging, to integrate model populations of cutaneous mechanoreceptors into finite element models (Kumar et al., 2015).

## Gaps in spinal cord models

### Fusimotor control of muscle spindles

Muscle spindles have long figured prominently in theories of sensorimotor control. The size and speed of their sensory axons, their numbers, and distributions, and their highly evolved structure and fusimotor control mechanisms all suggest that they are functionally critical. All of these elements are well-described in existing computational models (Mileusnic et al., 2006). The problem is that only sparse information is available about how their sensitivity is controlled by the fusimotor system during natural behaviors (Loeb, 1984; Prochazka, 1999; Taylor et al., 2000).

The fusimotor apparatus has undergone a huge elaboration in mammals, including specializations within various parts of the musculoskeletal system (Richmond et al., 1986). Servocontrol models of sensorimotor control originally emphasized the role of spindle afferents in the clinically prominent monosynaptic stretch reflex, but this represents only a tiny fraction of their central projections. Spindle afferents contribute to a multitude of oligosynaptic circuits in spinal and brainstem interneurons where they are combined with descending command signals. They are also the dominant source of information about posture and kinesthesia (Scott and Loeb, 1994; Gandevia, 1996), which are necessary for high-level planning and evaluation of motor strategies in cerebral cortex and cerebellum. Simplistic rules for fusimotor control have been hypothesized [e.g., alpha-gamma coactivation (Vallbo, 1974), optimal transducer programming (Loeb and Marks, 1985)] but these are speculations rather than known facts.

### Connectivity of spinal interneurons

Signals from the brain and sensory afferents are mixed in spinal interneurons that project to the alpha motoneurons (Pierrot-Deseilligny and Burke, 2005). As a result, the influence of brain activity on muscle contractions and movement depends substantially on the connectivity of spinal interneurons. In the past century, many neural pathways from sensory receptors (cutaneous and proprioceptive) to alpha motoneurons have been identified. These pathways include the monosynaptic Ia excitation of alpha motoneurons and polysynaptic pathways involving propriospinal, Renshaw, Ia, and Ib interneurons. The pathways were identified mainly through electrophysiology techniques by perturbing sensory signals from one muscle and investigating the timing of enhancement or depression of alpha motoneuron activity of the same muscle as well as muscles that were functional antagonists, synergists, or both (for a recent review, see Loeb, 2014). Neural pathways involving cutaneous receptors are poorly defined relative to those involving proprioceptors because it is difficult to stimulate a large group of afferents from a homogeneous type of cutaneous receptor. In electrophysiology experiments, the connectivity of cutaneous pathways is investigated typically by stimulating cutaneous nerves or patches of skin, which contain neurons from many different types of cutaneous receptors (Pierrot-Deseilligny and Burke, 2005). Through this method, it is not possible to distinguish the contribution of each type of receptor on the resulting alpha motoneuron activity. Information from these experiments, therefore, cannot be used to accurately predict the effects of physiological activity of the various cutaneous receptor afferents on interneuron or alpha motoneuron activity in the spinal cord.

Neural pathways from cutaneous receptors and proprioceptors to alpha motoneurons involving three synapses or more are not well-defined because current experimental techniques are limited. In electrophysiology experiments, if one of the neurons in the polysynaptic chain is hyperpolarized substantially via descending control, the whole chain will be invisible to the experimenter. Even if the effect on alpha motoneuron activity is observable, it will likely be highly variable and even differ in sign across subjects and experiments because descending control of each one of the neurons in the chain will likely depend strongly on the conditions of the experiment and physiological state of the subject. Furthermore, sensory afferent collaterals may activate several pathways in parallel with similar latencies, making them indistinguishable in the alpha motoneuron signal.

### Role of presynaptic modulation

Neurotransmitter release by the presynaptic neuron depends not only action potential rate, but also on the level of presynaptic inhibition/facilitation induced by interneurons forming axoaxonic connections with the presynaptic neurons. The activity of these interneurons are controlled by descending projections as well as by sensory afferents, but this connectivity is poorly understood (Rudomin and Schmidt, 1999). In theory, presynaptic control could allow selective gating of signals from different sensory modalities, muscles, and interneurons depending on the task. It is not clear, however, what portion of the presynaptic terminals the brain can control independently (Sirois et al., 2013). Furthermore, it is not clear how presynaptic input varies across tasks. There may be a set of presynaptic inputs that are useful for a wide range of tasks (see Fink et al., 2014), thereby reducing the number of control parameters the brain would have to learn to perform new movements.

## Status of brain-level models

Models of different parts of the brain tend to be abstract because of the lack of specific knowledge of the neural circuits that process signals from their input sources and the lack of knowledge of the specific neural circuits that their output projections influence. Spinal interneurons receive input from many parts of the brain, including cerebral cortex, brain stem, and tectum. The distribution of these inputs among spinal interneurons as well as the specific source locations are poorly understood. New tools for tracing and modulating connections based on genetic engineering of specific cell-types are just starting to reveal the functional relationships between brain and spinal cord circuits (Akay et al., 2014; Azim et al., 2014; Esposito et al., 2014).

### Cerebral cortex

Voluntary motor control is usually assumed to reside in a small set of frontal lobe cortical areas. Damage to these areas in humans such as from stroke results in profound losses of such behaviors. Top-down models often attribute to cortex most or all of the control function involved in learning and executing these behaviors. Cortex seems to be necessary for learning new motor skills but may not be sufficient (see below) or even necessary for their execution (Kawai et al., 2015).

It has long been known that the cerebral cortex is organized in layers that are associated with specific types of neurons, input sources and output destinations. These could provide the substrate for bottom-up modeling. However, little is known about the local connections among neurons from different layers and even less about the connectivity among the thalamus and distant cortical columns that span the many cortical areas involved in sensorimotor control (Hooks et al., 2013; Kaneko, 2013). For this reason, circuit models tend to rely heavily on correlations of activity between a very small subset of cortical neurons either in behaving animals and/or in response to electrical stimulation (Chadderdon et al., 2014). The apparent circuits derived from these studies apply only to the small number of neurons observed and may deviate substantially from true circuitry because there are many pathways with several interneurons between recording and stimulation sites; these interneurons may block activity through some pathways. Interneuron activity depends on experimental conditions so it is likely that the net excitatory/inhibitory influence observed during the highly constrained experiments does not apply to many sensorimotor behaviors. The specific output pathways to cerebellum and subcortical structures such as the brain stem nuclei, propriospinal interneurons, and segmental interneurons are also poorly understood.

The cerebral cortex has long been an attractive candidate for computational models because of its obvious importance for learning new tasks. The rise of digital logic in the 1940s and 50s coincided with the development of electrophysiological methods to study the activity of individual neurons, leading to the compelling notion of neurons as logical and gates (McCulloch and Pitts, 1943) and learning as rules for changing the weighting of the inputs (Hebb, 1949). Models of cortical function have mostly been elaborations of this basic scheme (Marr, 1970). The problem is that the principal output cells of the cortex appear to be vastly more complex in their computational functions. Each of the numerous tiny spines that extend from their dendrites appears to function as a sophisticated temporospatial signal processor whose output gain can be individually adjusted before contributing to the all-or-none output of the neuron as a whole (Polsky et al., 2004; Jadi et al., 2012). Ambitious attempts are underway to develop computational models of cerebral cortex based on exhaustive analysis of neural connectivity (Markram, 2006), but the computational algorithm for the individual cortical neurons is now less clear than was originally assumed.

### Basal ganglia—thalamus

The thalamus relays and processes information from the cerebral cortex, cerebellum, brain stem, and spinal cord, and its output neurons project to various areas of the cerebral cortex. Despite its important role, the interneuron connectivity of the thalamus and associated circuitry in the basal ganglia (Bosch-Bouju et al., 2013) is poorly understood and it is commonly omitted in models of sensorimotor control. Current models of this subsystem (Nelson and Kreitzer, 2014) are still based on simplistic analogies between neurons and electronic logic gates (McCulloch and Pitts, 1943), despite data demonstrating that their circuits and even individual neurons generate complex patterns of spontaneous activity (Llinas, 1988; Nakamura et al., 2014). Computational models of individual neurons with such properties are in their infancy. Incorporating them into large-scale systems requires many more parameters and assumptions about their individual properties and connectivity patterns, as well as much greater computing power.

### Midbrain tectum

The tectum (superior and inferior colliculus in mammals) may play a much larger role in sensorimotor interaction with external objects than is generally acknowledged. The superior colliculus has been intensively studied and modeled for its role in directing saccadic eye movements to visual stimuli (Fecteau and Munoz, 2006). The inferior colliculus has been studied mostly as the “relay” in which sound localization information from both ears is conveyed to the auditory cortex (Slee and Young, 2014). But in fish and amphibia, the analogous subsystem controls most of the purposeful motor behaviors of the organisms, which must be fast, accurate and well-coordinated. In all vertebrates, all exteroceptive senses capable of providing localization information about an external object (vision, hearing, and touch) converge on the tectum. The tectal outputs project to brainstem nuclei and spinal cord layers that control all of the muscles required to acquire such targets, whether by saccadic gaze movements of eye and head (and auricular pinnae in most species other than primates) or reaching movements of the limbs (and jaws and tongue in many species) (Saitoh et al., 2007; Kozlov et al., 2014; Philipp and Hoffmann, 2014). The direct projections from retina to superior colliculus are known to be the source of express saccades (Munoz and Wurtz, 1992), accurately directed gaze movements that can acquire targets about twice as fast as the transcortical loop (lateral geniculate to occipital cortex and frontal eye fields) that actually projects back to the superior colliculus, which appears to control the metrics of the saccade. There have been several reports of extremely short latency corrections of reaching movements to visual targets that shift position (Gribble et al., 2002; Wilmut et al., 2006; Perfiliev et al., 2010) and in avoidance of obstacles to locomotion (Weerdesteyn et al., 2004), which would be consistent with the tectum performing the same function in limb movements as in gaze movements. If true, this would affect profoundly the assumptions made about the computational tasks of the motor and parietal cortical regions associated with reaching in extrapersonal space, perhaps the most common task for which top-down models have been constructed.

### Cerebellum and brainstem

The cerebellum makes an excellent poster child for the problem of modeling any one of the many subsystems that subserve sensorimotor function. Its cytoarchitecture and neurophysiology is highly distinctive, relatively simple and homogeneous and better documented than any other part of the central nervous system. It is important enough to have been endowed with over half of the neurons in almost any vertebrate. Lesions to the adult cerebellum produce profound and distinctive motor deficits, yet humans born with substantial cerebellar atresia function surprisingly well (Walker, 1944). The cerebellum has attracted several detailed computational models of its function (Albus, 1975; Marr and Thach, 1991; Kawato and Gomi, 1993; Kawato and Samejima, 2007), yet it can be plausibly argued to subserve either motor (Thach, 2014) or sensory (Bower, 1997) function, to the virtual exclusion of the other.

Although the neurons of the cerebellum and their connectivity are understood better than most structures in the brain, the precise input sources and their target neurons as well as the precise destinations of the output projections are not well-known. For example, the climbing fibers from the inferior olivary nucleus in the medulla provide a substantial portion of the input to the cerebellum, but little is known about the inputs to the inferior olive and how they are processed. Climbing fiber activity is known to be influenced by the reticular formation and red nucleus of the brain stem, several areas from the cerebral cortex, as well as by signals from the periphery related to proprioception and touch (Brown et al., 1977; Stecina et al., 2013). Specific sources of these signals, how they are processed in the inferior olive, and how the processed output affects cerebellar function is not known. Another major source of input to the cerebellum is the mossy fiber projections from the lateral reticular nucleus of the reticular formation. There is a large degree of convergence of afferent activity ascending from the spinal cord with activity descending from the cerebral cortex, tectum, red nucleus and other parts of the brain stem (Alstermark and Ekerot, 2013). The precise source of these inputs as well as the interneuronal circuits that integrate them is largely unknown.

The brain stem possesses many subdivisions including the inferior olive, vestibular nuclei, reticular formation and red nucleus with distinct set of inputs, outputs, and interneurons. Although they have an important functional role for even simple reach and grasp movements (Alstermark and Isa, 2012), the connectivity of their neural circuits as well as the precise location of their inputs and outputs are poorly understood (Kennedy, 1990; Kuchler et al., 2002). Much of the output of cerebellum is targeted to these structures, which also receive the proprioceptive and vestibular sensory signals required to coordinate movements across the entire body. Most studies of motor behavior focus on the “prime mover” muscles most closely associated with the task, but the freely mobile, multiarticulated structures of most terrestrial animals are actually quite difficult to stabilize because of intersegmental Coriolis forces (Lackner and DiZio, 2000). For example, human subjects asked to flex and extend their elbow tend to keep the wrist still without thinking, whereas a similar weight connected by a hinge to a stick being wagged up and down would flop uncontrollably. This problem could be solved by simply cocontracting the muscles of the wrist to stiffen the joint, but that would be energetically inefficient. Instead, subjects deftly time the activation of the many different wrist muscles to cancel the rapidly changing Coriolis forces. This problem is greatly magnified when trying to maintain balance of the whole body during any rapid movement of any body part, a problem that is constantly changing as the musculoskeletal system develops and ages. Perhaps the best studied model for adaptive tuning of stabilizing reflex gains is the vestibulo-ocular reflex, which involves changes in brainstem nuclei mediated by cerebellar plasticity (Kawato and Gomi, 1992; Raymond et al., 1996; Clopath et al., 2014). The details of these circuits and their learning rules remain contentious and it is uncertain if they generalize to other sensorimotor behaviors.

## Use of systems models to demonstrate competency

Many anatomical structures of the sensorimotor system have been investigated to characterize their inputs, outputs, and intermediate processing (although as explained above, many important knowledge gaps remain). Their complexity often makes it impossible to intuit their input-output transformation. Neural networks within these structures, for example, have a large number of neurons with various non-linear properties and substantial convergent, divergent, and recurrent connections that make it difficult to infer how they process a set of incoming signals. Computational models are therefore essential for predicting these non-intuitive interactions and provide insight into the possible transformations that a neural network can apply to a range of input signal patterns. Intuition is even less useful for predicting the collective input-output transformation of a system of interconnected neural networks formed by distinct anatomical structures in the nervous system such as those shown in Figure 1. Understanding such complex interactions is necessary for determining the relative roles of these anatomical structures in sensorimotor control and ultimately unraveling the mechanisms involved.

The sparse knowledge of the anatomy and physiology underlying the sensorimotor system precludes a complete understanding of the mechanism of sensorimotor control. This knowledge, however, can be exploited to understand the competency of the known properties and suggest experimental investigations for furthering our understanding and updating the models. It can be tested, for example, if a set of known properties of the sensorimotor system is sufficient for generating specific behaviors. If the properties are sufficient for generating a particular behavior then this would suggest that these properties may play a significant role. It has been shown recently that the known spinal circuits described above are sufficient for generating the muscle dynamics of wrist (Raphael et al., 2010) and arm movement (Tsianos et al., 2011, 2014), which has been traditionally assumed to arise largely from commands issued by the motor cortex. These results emphasize the possibility that spinal circuits can make large contributions to voluntary movement and encourage further experimental testing. It has also been shown that descending commands to models of known spinal circuitry can be linearly interpolated to generate intermediate movements (Tsianos et al., 2014). Such simple interpolation along with the modeled musculoskeletal system and spinal circuitry properties were sufficient to reproduce the extent of learning generalization observed experimentally for similar tasks. This result suggests that much of the generalization of learning observed experimentally may arise from simple combinations of learned voluntary commands rather than through the use of internal inverse models of the sensorimotor system. This is consistent with the tendency of human subjects to adapt to changes in the musculoskeletal system by relatively simple scaling of the original motor programs rather than computation of new programs that offered superior performance (de Rugy et al., 2012a,b).

## Use of systems models to prove insufficiency

If modeled properties of the sensorimotor system are not sufficient to explain a particular behavior, then these properties must interact with other biological properties that are either not modeled or not known. Other known properties can be added to the model incrementally to test which configurations are sufficient for reproducing the desired behavior. For example, activation of muscles and feedback through spinal circuits contribute to stable movements due to the relatively short time delays involved; however, the contribution of spinal circuits is limited for whole body tasks that require muscle coordination between distant body parts. Spinal circuits coordinate activity among a limited number of adjacent muscles and joints because inputs to a given spinal interneuron originate from only a few adjacent spinal segments. There are many relatively fast circuits involving anatomical structures rostral to the spinal cord such as the brain stem (Esposito et al., 2014), tectum (Philipp and Hoffmann, 2014), cerebellum (Azim et al., 2014) and even sensorimotor cortex (Scott, 2004, 2012; Nashed et al., 2015) that receive afferent signals from throughout the body and may therefore contribute to the dynamics and stability of such movement. Known circuits from these anatomical structures can be incorporated in models to test their sufficiency, which would depend on their specific connectivity as well as the range of input sources and their extent of convergence. If multiple anatomical structures appear sufficient, then the modeled task could be varied systematically to search for situations where each one becomes uniquely competent. This would indicate the types of sensorimotor tasks and conditions that each anatomical structure might subserve in the biological system. If known properties of a given anatomical structure cannot account for the behavior, it could be that the model for the structure is inadequate or that other structures are required. Such negative results are particularly valuable in identifying opportunities to advance understanding of the system as a whole.

## General obstacles to successful modeling

It is always easy to end a scientific paper with a call for more experiments to provide more data to fill in the missing pieces of knowledge. This brief review has pointed out many places where basic knowledge about neural connectivity is insufficient to permit reductionist modeling. But there are also many places where the connectivity is relatively well-known and detailed models have been constructed, only to expose contentious disagreements about what role the structure actually plays in a given behavior.

As David Marr pointed out, models must start with a top-level theory of computation—a division of function into a sequence or hierarchy of tasks (Marr, 1982). Only then can one contemplate the computational algorithms that might subserve each of those tasks and, below that, the machinery that performs each algorithm. The physiology and connectivity of individual neurons provides information about the machinery level, leaving the modeler to guess about the tasks and the algorithms.

It used to be feasible for a researcher to become familiar with most of the literature about most of the CNS and to construct a systems level model of behaviors that considered both its phylogenetic and ontological origins; for example, (Ayres, 1975). Most researchers now spend a lifetime learning the details of and formulating hypotheses about one or two of the many subsystems that somehow contribute to sensorimotor behaviors. They tend naturally to assume that the subsystem that they are studying is primarily responsible for the behaviors that they employ in their experimental designs. Their models of “their” subsystem must nevertheless be reconciled with some current dogma about the role of the other subsystems, as promulgated by other researchers with equally narrow and parochial views.

“It was six men of Indostan To learning much inclined, Who went to see the Elephant (Though all of them were blind), That each by observation Might satisfy his mind…

And so these men of Indostan Disputed loud and long, Each in his own opinion Exceeding stiff and strong, Though each was partly in the right, And all were in the wrong!”

*—From “Blind Men and the Elephant” by John Godfrey Saxe (1816–1887)*.

### Conflict of interest statement

The authors declare that the research was conducted in the absence of any commercial or financial relationships that could be construed as a potential conflict of interest.

## References

[B1] Akay T. Tourtellotte W. G. Arber S. Jessell T. M. (2014). Degradation of mouse locomotor pattern in theabsence of proprioceptive sensory feedback. Proc. Natl. Acad. Sci. U.S.A. 111, 16877–16882. 10.1073/pnas.1419045111 25389309 PMC4250167

[B2] Albus J. S. (1975). A new approach to manipulator control: the cerebellar model articulation controller (CMAC). J. Dyn. Sys. Meas. Control 97, 220–227. 10.1115/1.3426922

[B3] Alstermark B. Ekerot C. (2013). The lateral reticular nucleus: a precerebellar centre providing the cerebellum with overview and integration of motor functions at systems level. A new hypothesis. J. Physiol. 591, 5453–5458. 10.1113/jphysiol.2013.256669 24042498 PMC3853488

[B4] Alstermark B. Isa T. (2012). Circuits for skilled reaching and grasping. Annu. Rev. Neurosci. 35, 559–578. 10.1146/annurev-neuro-062111-150527 22524789

[B5] d'Avella A. Portone A. Fernandez L. Lacquaniti F. (2006). Control of fast-reaching movements by muscle synergy combinations. J. Neurosci. 26, 7791–7810. 10.1523/JNEUROSCI.0830-06.2006 16870725 PMC6674215

[B6] Ayres A. J. (1975). Sensorimotor foundations of academic ability, in Perceptual and Learning Disabilities in Children, Vol 2: Research and Theory, eds Cruickshank W. M. Hallahan D. P. (New York, NY: Syracuse University Press), 300–360.

[B7] Azim E. Jiang J. Alstermark B. Jessell T. M. (2014). Skilled reaching relies on a V2a propriospinal internal copy circuit. Nature 508, 357–363. 10.1038/nature13021 24487617 PMC4230338

[B8] Baraduc P. Wolpert D. M. (2002). Adaptation to a visuomotor shift depends on the starting posture. J. Neurophysiol. 88, 973–981. Available online at: http://jn.physiology.org/content/88/2/973.long 12163546 10.1152/jn.2002.88.2.973

[B9] Berniker M. Franklin D. W. Flanagan R. Wolpert D. M. Kording K. (2014). Motor learning of novel dynamics is not represented in a single global coordinate system: evaluation of mixed coordinate representations and local learning. J. Neurophysiol. 111, 1165–1182. 10.1152/jn.00493.2013 24353296 PMC3949315

[B10] Bernstein N. A. (1967). Human Motor Actions: Bernstein Reassessed (Translation, edited by Whiting H. T. A.). Oxford: Elsevier.

[B11] Bosch-Bouju C. Hyland B. I. Parr-Brownlie L. C. (2013). Motor thalamus integration of cortical, cerebellar and basal ganglia information: implications for normal and parkinsonian conditions. Front. Comput. Neurosci. 7:163. 10.3389/fncom.2013.00163 24273509 PMC3822295

[B12] Bower J. M. (1997). Is the cerebellum sensory for motor's sake, or motor for sensory's sake: the view from the whiskers of a rat? Prog. Brain Res. 114, 483–516. 9193161 10.1016/s0079-6123(08)63381-6

[B13] Brayanov J. B. Press D. Z. Smith M. A. (2012). Motor memory is encoded as a gain-field combination of intrinsic and extrinsic action representations. J. Neurosci. 32, 14951–14965. 10.1523/JNEUROSCI.1928-12.2012 23100418 PMC3999415

[B14] Brown I. E. Liinamaa T. L. Loeb G. E. (1996). Relationships between range of motion, L0, and passive force in five strap-like muscles of the feline hind limb. J. Morphol. 230, 69–77. 8843689 10.1002/(SICI)1097-4687(199610)230:1<69::AID-JMOR6>3.0.CO;2-I

[B15] Brown J. T. Chan-Palay V. Palay S. L. (1977). A study of afferent input to the inferior olivary complex in the rat by retrograde axonal transport of horseradish peroxidase. J. Comp. Neurol. 176, 1–22. 10.1002/cne.901760102 903429

[B16] Chadderdon G. L. Mohan A. Suter B. A. Neymotin S. A. Kerr C. C. Francis J. T.. (2014). Motor cortex microcircuit simulation based on brain activity mapping. Neural Comput. 26, 1239–1262. 10.1162/NECO_a_00602 24708371 PMC4887269

[B17] Clopath C. Badura A. de Zeeuw C. I. Brunel N. (2014). A cerebellar learning model of vestibulo-ocular reflex adaptation in wild-type and mutant mice. J. Neurosci. 34, 7203–7215. 10.1523/JNEUROSCI.2791-13.2014 24849355 PMC6608186

[B18] Coelho C. J. Przybyla A. Yadav V. Sainburg R. L. (2013). Hemispheric differences in the control of limb dynamics: a link between arm performance asymmetries and arm selection patterns. J. Neurophysiol. 109, 825–838. 10.1152/jn.00885.2012 23155169 PMC3567394

[B19] Crawford G. L. Horowits R. (2011). Scaffolds and chaperones in myofibril assembly: putting the striations in striated muscle. Biophys. Rev. 3, 25–32. 10.1007/s12551-011-0043-x 21666840 PMC3110075

[B20] Crevecoeur F. Scott S. H. (2014). Beyond muscles stiffness: importance of state-estimation to account for very fast motor corrections. PLoS. Comp. Biol. 10:e1003869. 10.1371/journal.pcbi.1003869 25299461 PMC4191878

[B21] de Rugy A. Davoodi R. Carroll T. J. (2012a). Changes in wrist muscle activity with forearm posture: implications for the study of sensorimotor transformations. J. Neurophysiol. 108, 2884–2895. 10.1152/jn.00130.2012 22972965

[B22] de Rugy A. Loeb G. E. Carroll T. J. (2012b). Muscle coordination is habitual rather than optimal. J. Neurosci. 32, 7384–7391. 10.1523/JNEUROSCI.5792-11.2012 22623684 PMC6622296

[B23] Enoka R. M. Duchateau J. (2008). Muscle fatigue: what, why and how it influences muscle function. J. Physiol. 586, 11–23. 10.1113/jphysiol.2007.139477 17702815 PMC2375565

[B24] Esposito M. S. Capelli P. Arber S. (2014). Brainstem nucleus MdV mediates skilled forelimb motor tasks. Nature 508, 351–356. 10.1038/nature13023 24487621

[B25] Fecteau J. H. Munoz D. P. (2006). Salience, relevance, and firing: a priority map for target selection. Trends Cogn. Sci. (Regul. Ed.) 10, 382–390. 10.1016/j.tics.2006.06.011 16843702

[B26] Fink A. J. P. Croce K. R. Huang J. Abbott L. F. Jessell T. M. Azim E. (2014). Presynaptic inhibition of spinal sensory feedback ensures smooth movement. Nature 509, 43–48. 10.1038/nature13276 24784215 PMC4292914

[B27] Gandevia S. C. (1996). Kinesthesia: roles for afferent signals and motor commands, in Handbook of Physiology Section 12, eds Rowell L. B. Shepherd J. T. (London: Oxford University Press), 128–172.

[B28] Gandolfo F. Mussa-Ivaldi F. A. Bizzi E. (1996). Motor learning by field approximation. Proc. Natl. Acad. Sci. U.S.A. 93, 3843–3846. 10.1073/pnas.93.9.3843 8632977 PMC39446

[B29] Gribble P. L. Everling S. Ford K. Mattar A. (2002). Hand-eye coordination for rapid pointing movements. Exp. Brain Res. 145, 372–382. 10.1007/s00221-002-1122-9 12136387

[B30] Grigg P. Hoffman A. H. (1984). Ruffini mechanoreceptors in isolated joint capsule: responses correlated with strain energy density. Somatosens. Res. 2, 149–162. 6528149 10.1080/07367244.1984.11800555

[B31] Hebb D. O. (1949). The Organization of Behavior. New York, NY: Wiley.

[B32] Hogan N. (1984). An organising principle for a class of voluntary movements. J. Neurosci. 4, 2745–2754. 6502203 10.1523/JNEUROSCI.04-11-02745.1984 PMC6564718

[B33] Hooks B. M. Mao T. Gutnisky D. A. Yamawaki N. Svoboda K. Shepherd G. M. (2013). Organization of cortical and thalamic input to pyramidal neurons in mouse motor cortex. J. Neurosci. 33, 748–760. 10.1523/JNEUROSCI.4338-12.2013 23303952 PMC3710148

[B34] Jadi M. Polsky A. Schiller J. Mel B. W. (2012). Location-Dependent Effects of Inhibition on Local Spiking in Pyramidal Neuron Dendrites. PLoS Comput. Biol. 8:e1002550. 10.1371/journal.pcbi.1002550 22719240 PMC3375251

[B35] Kaneko T. (2013). Local connections of excitatory neurons in motor-associated cortical areas of the rat. Front. Neural Circuits 7:75. 10.3389/fncir.2013.00075 23754982 PMC3664775

[B36] Kawai R. Markman T. Poddar R. Ko R. Fantana Antoniu L. Dhawale Ashesh K.. (2015) Motor cortex is required for learning but not for executing a motor skill. Neuron 86, 800–812. 10.1016/j.neuron.2015.03.024 25892304 PMC5939934

[B37] Kawato M. Gomi H. (1992). The cerebellum and VOR/OKR learning models. Trends Neurosci. 15, 445–453. 10.1016/0166-2236(92)90008-V 1281352

[B38] Kawato M. Gomi H. (1993). Feedback-error-learning model of cerebellar motor control, in Role of the Cerebellum and Basal Ganglia in Voluntary Movement, eds Mano N. Hamada I. DeLong M. R. (Amsterdam: Elsevier), 51–61.

[B39] Kawato M. Samejima K. (2007). Efficient reinforcement learning: computational theories, neuroscience and robotics. Curr. Opin. Neurobiol. 17, 205–212. 10.1016/j.conb.2007.03.004 17374483

[B40] Kennedy P. R. (1990). Corticospinal, rubrospinal and rubro-olivary projections: a unifying hypothesis. Trends Neurosci. 13, 474–479. 10.1016/0166-2236(90)90079-P 1703677

[B41] Kozlov A. K. Kardamakis A. A. Kotaleski J. H. Grillner S. (2014). Gating of steering signals through phasic modulation of reticulospinal neurons during locomotion. Proc. Natl. Acad. Sci. U.S.A. 111, 3591–3596. 10.1073/pnas.1401459111 24550483 PMC3948313

[B42] Krakauer J. W. Mazzoni P. Ghazizadeh A. Ravindran R. Shadmehr R. (2006). Generalization of motor learning depends on the history of prior action. PLoS Biol. 4:e316. 10.1371/journal.pbio.0040316 16968135 PMC1563496

[B43] Krakauer J. W. Pine Z. M. Ghilardi M. Ghez C. (2000). Learning of visuomotor transformations for vectorial planning of reaching trajectories. J. Neurosci. 20, 8916–8924. Available online at: http://www.jneurosci.org/content/20/23/8916.long 11102502 10.1523/JNEUROSCI.20-23-08916.2000 PMC6773094

[B44] Kuchler M. Fouad K. Weinmann O. Schwab M. E. Raineteau O. (2002). Red nucleus projections to distinct motor neuron pools in the rat spinal cord. J. Comp. Neurol. 448, 349–359. 10.1002/cne.10259 12115698

[B45] Kumar S. Liu G. Schloerb D. W. Srinivasan M. A. (2015). Viscoelastic characterization of the primate finger pad *in vivo* by microstep indentation and three-dimensional finite element models for tactile sensation studies. J. Biomech. Eng. 137, 061002. 10.1115/1.4029985 25751365 PMC4403516

[B46] Lackner J. R. DiZio P. A. (2000). Aspects of body self-calibration. Trends Cogn. Sci. (Regul. Ed.) 4, 279–288. 10.1016/S1364-6613(00)01493-5 10859572

[B47] Llinas R. R. (1988). The intrinsic electrophysiological properties of mammalian neurons: insights into central nervous system function. Science 242, 1654–1664. 10.1126/science.3059497 3059497

[B48] Loeb G. E. (1984). The control and responses of mammalian muscle spindles during normally executed motor tasks. Exerc. Sport Sci. Rev. 12, 157–204. 10.1249/00003677-198401000-00008 6234174

[B49] Loeb G. E. (2012). Optimal isn't good enough. Biol. Cybern. 106, 757–765. 10.1007/s00422-012-0514-6 22895830

[B50] Loeb G. E. (2014). Spinal cord, integrated (Non CPG) models of, in Encyclopedia of Computational Neuroscience, eds Jaeger D. Jung R. (New York, NY: Springer), 1–13.

[B51] Loeb G. E. Brown I. E. Lan N. Davoodi R. (2002). The importance of biomechanics. Adv. Exp. Med. Biol. 508, 481–487. 10.1007/978-1-4615-0713-0_54 12171146

[B52] Loeb G. E. Marks W. B. (1985). Optimal control principles for sensory transducers, in Proceedings of the International Symposium: The Muscle Spindle, eds Boyd I. A. Gladden M. H. (London: MacMillan Ltd), 409–415.

[B53] Markram H. (2006). The blue brain project. Nat. Rev. Neurosci. 7, 153–160. 10.1038/nrn1848 16429124

[B54] Marr D. (1970). A theory for cerebral neocortex. Proc. Philos. Trans. R. Soc. 176, 161–234. 10.1098/rspb.1970.0040 4394740

[B55] Marr D. (1982). Vision: A Computational Investigation into the Human Representation and Processing of Visual Information. New York, NY: Henry Holt and Co., Inc.

[B56] Marr D. Thach W. T. (1991). A theory of cerebellar cortex, in From the Retina to the Neocortex, ed Vaina L. (Boston, MA: Springer), 11–50.

[B57] Mattar A. A. G. Ostry D. J. (2007). Modifiability of generalization in dynamics learning. J. Neurophysiol. 98, 3321–3329. 10.1152/jn.00576.2007 17928561

[B58] Mattar A. A. G. Ostry D. J. (2010). Generalization of dynamics learning across changes in movement amplitude. J. Neurophysiol. 104, 426–438. 10.1152/jn.00886.2009 20463200 PMC2904213

[B59] McCulloch W. S. Pitts W. (1943). A logical calculus of the ideas imminent in nervous activity. Bull. Mathem. Biophys. 5, 115–133. 10.1007/BF02478259 2185863

[B60] Mileusnic M. P. Brown I. E. Lan N. Loeb G. E. (2006). Mathematical models of proprioceptors. I. Control and transduction in the muscle spindle. J. Neurophysiol. 96, 1772–1788. 10.1152/jn.00868.2005 16672301

[B61] Munoz D. P. Wurtz R. H. (1992). Role of the rostral superior colliculus in active visual fixation and execution of express saccades. J. Neurophysiol. 67, 1000–1002. 1588382 10.1152/jn.1992.67.4.1000

[B62] Nakamura K. C. Sharott A. Magill P. J. (2014). Temporal coupling with cortex distinguishes spontaneous neuronal activities in identified basal ganglia-recipient and cerebellar-recipient zones of the motor thalamus. Cereb. Cortex 24, 81–97. 10.1093/cercor/bhs287 23042738 PMC3862266

[B63] Nashed J. Y. Kurtzer I. L. Scott S. H. (2015). Context-dependent inhibition of unloaded muscles during the long-latency epoch. J. Neurophysiol. 113, 192–202. 10.1152/jn.00339.2014 25274342

[B64] Nelson A. B. Kreitzer A. C. (2014). Reassessing models of basal ganglia function and dysfunction. Annu. Rev. Neurosci. 37, 117–135. 10.1146/annurev-neuro-071013-013916 25032493 PMC4416475

[B65] Pearson T. S. Krakauer J. W. Mazzoni P. (2010). Learning not to generalize: modular adaptation of visuomotor gain. J. Neurophysiol. 103, 2938–2952. 10.1152/jn.01089.2009 20357068 PMC2888232

[B66] Perfiliev S. Isa T. Johnels B. Steg G. Wessberg J. (2010). Reflexive limb selection and control of reach direction to moving targets in cats, monkeys, and humans. J. Neurophysiol. 104, 2423–2432. 10.1152/jn.01133.2009 20810693

[B67] Philipp R. Hoffmann K. (2014). Arm movements induced by electrical microstimulation in the superior colliculus of the macaque monkey. J. Neurosci. 34, 3350–3363. 10.1523/JNEUROSCI.0443-13.2014 24573292 PMC6795300

[B68] Pierrot-Deseilligny E. Burke D. C. (2005). The Circuitry of the Human Spinal Cord: Its Role in Motor Control and Movement Disorders. Cambridge: Cambridge University Press.

[B69] Polsky A. Mel B. W. Schiller J. (2004). Computational subunits in thin dendrites of pyramidal cells. Nat. Neurosci. 7, 621–627. 10.1038/nn1253 15156147

[B70] Prochazka A. (1999). Quantifying proprioception. Prog. Brain Res. 123, 133–142. 10635710

[B71] Raphael G. Tsianos G. A. Loeb G. E. (2010). Spinal-Like regulator facilitates control of atwo-degree-of-freedom wrist. J. Neurosci. 30, 9431–9444. 10.1523/JNEUROSCI.5537-09.2010 20631172 PMC6632449

[B72] Raymond J. L. Lisberger S. G. Mauk M. D. (1996). The cerebellum: a neuronal learning machine? Science 272, 1126–1131. 10.1126/science.272.5265.1126 8638157

[B73] Richmond F. J. R. Bakker G. J. Bakker D. A. Stacey M. J. (1986). The innervation of tandem muscle spindles in the cat neck. J. Comp. Neurol. 245, 483–497. 10.1002/cne.902450405 2422225

[B74] Richmond F. J. R. Singh K. Corneil B. D. (2001). Neck muscles in the rhesus monkey. I. muscle morphometry and histochemistry. J. Neurophysiol. 86, 1717–1728. Available online at: http://jn.physiology.org/content/86/4/1717 11600634 10.1152/jn.2001.86.4.1717

[B75] Rudomin P. Schmidt R. F. (1999). Presynaptic inhibition in the vertebrate spinal cord revisited. Exp. Brain Res. 129, 1–37. 10.1007/s002210050933 10550500

[B76] Saitoh K. Ménard A. Grillner S. (2007). Tectal control of locomotion, steering, and eye movements in lamprey. J. Neurophysiol. 97, 3093–3108. 10.1152/jn.00639.2006 17303814

[B77] Schieber M. H. Santello M. (2004). Hand function: peripheral and central constraints on performance. J. Appl. Physiol. 96, 2293–2300. 10.1152/japplphysiol.01063.2003 15133016

[B78] Scott S. H. (2004). Optimal feedback control and the neural basis of volitional motor control. Nat. Rev. Neurosci. 5, 532–546. 10.1038/nrn1427 15208695

[B79] Scott S. H. (2012). The computational and neural basis of voluntary motor control and planning. Trends Cogn. Sci. (Regul. Ed.) 16, 541–549. 10.1016/j.tics.2012.09.008 23031541

[B80] Scott S. H. Loeb G. E. (1994). The computation of position sense from spindles in mono-and multiarticular muscles. J. Neurosci. 14, 7529–7540. 7996193 10.1523/JNEUROSCI.14-12-07529.1994 PMC6576884

[B81] Shadmehr R. Mussa-Ivaldi A. (1994). Adaptive representation of dynamics during learning of a motor task. J. Neurosci. 14, 3208–3224. 8182467 10.1523/JNEUROSCI.14-05-03208.1994 PMC6577492

[B82] Sirois J. Frigon A. Gossard J. (2013). Independent control of presynaptic inhibition by reticulospinal and sensory inputs at rest and during rhythmic activities in the cat. J. Neurosci. 33, 8055–8067. 10.1523/JNEUROSCI.2911-12.2013 23637195 PMC6618948

[B83] Slee S. J. Young E. D. (2014). Alignment of sound localization cues in the nucleus of the brachium of the inferior colliculus. J. Neurophysiol. 111, 2624–2633. 10.1152/jn.00885.2013 24671535 PMC4044433

[B84] Stecina K. Fedirchuk B. Hultborn H. (2013). Information to cerebellum on spinal motor networks mediated by the dorsal spinocerebellar tract. J. Physiol. (Lond.) 591, 5433–5443. 10.1113/jphysiol.2012.249110 23613538 PMC3853486

[B85] Taylor A. Durbaba R. Ellaway P. H. Rawlinson S. (2000). Patterns of fusimotor activity during locomotion in the decerebrate cat deduced from recordings from hindlimb muscle spindles. J. Physiol. 522, 515–532. 10.1111/j.1469-7793.2000.t01-3-00515.x 10713974 PMC2269771

[B86] Thach W. (2014). Does the cerebellum initiate movement? Cerebellum 13, 139–150. 10.1007/s12311-013-0506-7 23964018

[B87] Trotter J. A. (1993). Functional morphology of force transmission in skeletal muscle. Acta Anat. 146, 205–222. 10.1159/000147459 8317197

[B88] Tsianos G. A. Goodner J. Loeb G. E. (2014). Useful properties of spinal circuits for learning and performing planar reaches. J. Neural Eng. 11:056006. 10.1088/1741-2560/11/5/056006 25082652

[B89] Tsianos G. A. Raphael G. Loeb G. E. (2011). Modeling the potentiality of spinal-like circuitry for stabilization of a planar arm system. Prog. Brain Res. 194, 203–213. 10.1016/b978-0-444-53815-4.00006-6 21867805

[B90] Tsianos G. A. Rustin C. Loeb G. E. (2012). Mammalian muscle model for predicting force and energetics during physiological behaviors. IEEE Trans. Neural Syst. Rehabil. Eng. 20, 117–133. 10.1109/TNSRE.2011.2162851 21859633

[B91] Vallbo A. B. (1974). Human muscle spindle discharge during isometric voluntary contractions. Amplitude relations between spindle frequency and torque. Acta Physiol. Scand. 90, 319–336. 10.1111/j.1748-1716.1974.tb05594.x 4274638

[B92] van der Helm F. C. (1994). A finite element musculoskeletal model of the shoulder mechanism. J. Biomech. 27, 551–569. 10.1016/0021-9290(94)90065-5 8027090

[B93] Walker A. E. (1944). A case of congenital atresia of the foramina of luschka and magendie: surgical cure. J. Neuropathol. Exp. Neurol. 3, 368–373. 10.1097/00005072-194410000-00004

[B94] Weerdesteyn V. Nienhuis B. Hampsink B. Duysens J. (2004). Gait adjustments in response to an obstacle are faster than voluntary reactions. Hum. Mov. Sci. 23, 351–363. 10.1016/j.humov.2004.08.011 15541522

[B95] Wilmut K. Wann J. Brown J. (2006). How active gaze informs the hand in sequential pointing movements. Exp. Brain Res. 175, 654–666. 10.1007/s00221-006-0580-x 16794847

